# On the choice of methodology for evaluating dose-rate effects on radiation-related cancer risks

**DOI:** 10.1007/s00411-021-00920-y

**Published:** 2021-06-25

**Authors:** Linda Walsh, Roy Shore, Tamara V. Azizova, Werner Rühm

**Affiliations:** 1 Department of Physics, Science Faculty, University of Zürich, Winterthurerstrasse 190, 8057 Zürich, Switzerland; 2 Department of Population Health, New York University Grossman School of Medicine, New York, USA; 3 Southern Urals Biophysics Institute, Ozyorskoe shosse 19, Ozyorsk, Chelyabinsk region 456780 Russia; 4 Institute of Radiation Medicine, Helmholtz Zentrum München- German Research Center for Environmental Health, 85764 Neuherberg, Germany

**Keywords:** Dose-rate effects, Meta-analysis, Radiation cancer risk

## Abstract

Recently, several compilations of individual radiation epidemiology study results have aimed to obtain direct evidence on the magnitudes of dose-rate effects on radiation-related cancer risks. These compilations have relied on meta-analyses of ratios of risks from low dose-rate studies and matched risks from the solid cancer Excess Relative Risk models fitted to the acutely exposed Japanese A-bomb cohort. The purpose here is to demonstrate how choices of methodology for evaluating dose-rate effects on radiation-related cancer risks may influence the results reported for dose-rate effects. The current analysis is intended to address methodological issues and does not imply that the authors recommend a particular value for the dose and dose-rate effectiveness factor. A set of 22 results from one recent published study has been adopted here as a test set of data for applying the many different methods described here, that nearly all produced highly consistent results. Some recently voiced concerns, involving the recalling of the well-known theoretical point—the ratio of two normal random variables has a theoretically unbounded variance—that could potentially cause issues, are shown to be unfounded when aimed at the published work cited and examined in detail here. In the calculation of dose-rate effects for radiation protection purposes, it is recommended that meta-estimators should retain the full epidemiological and dosimetric matching information between the risks from the individual low dose-rate studies and the acutely exposed A-bomb cohort and that a regression approach can be considered as a useful alternative to current approaches.

## Introduction

Many studies on radiation-related detrimental health effects rely on collecting results from published papers with the aim of providing compiled information to guide radiation protection. Information on how dose-rate effects may influence radiation-related cancer risks may be compiled from many modern radiation epidemiology studies. Such compilations of individual study results may be combined to provide evidence of whether or not the cancer risk per unit dose of chronic exposures accumulated over an extended period of time differs noticeably from the cancer risk per unit dose from a single acute exposure. The dose and dose-rate effectiveness factor (DDREF) concept (ICRP 1991) provides a general estimate of the ratio of cancer risk per unit of acute exposure to the cancer risk per unit of chronic exposure while concurrently aiming to provide estimates of extrapolation from high to low doses. DDREF is a radiation protection concept and may be interpreted as a combination of a low-dose effectiveness factor (LDEF) to extrapolate from high to low doses, and a dose-rate effectiveness factor (DREF) to extrapolate from high to low dose rates. In the currently recommended dose limits for occupational exposures (ICRP 2007), the assumption is that solid cancer risk factors are a factor of two lower than for the A-bomb survivors (i.e., DDREF = 2.0). However, ICRP is currently reviewing the usefulness of this concept and the weight of evidence for various numerical values of estimates for DDREF (Rühm et al. 2015, 2016; Shore et al. 2017).

Recently, several compilations of individual study results have aimed to obtain direct evidence on the magnitudes of the DREF. The purpose here is not to review all such studies, but rather to demonstrate how choices of methodology for evaluating dose-rate effects on radiation-related cancer risks may influence the DREF results reported. Two recent compilations, Jacob et al. (2009), Shore et al. (2017), applying very similar methodology, have been well cited and attracted positive feedback. However, concern also has been raised that the methods used in deriving the DREF results might require improvement (Little et al. 2021). A key concern about the methods is a well-known theoretical point (Stuart and Ord 1994): the ratio of two normal random variables has a theoretically unbounded variance, which in certain circumstances could yield practical inconsistencies in calculated variances, if this is not accounted for in a practical way. This could potentially be relevant, because the two studies, Jacob et al. (2009), Shore et al. (2017), produced meta-analysis estimates involving ratios of Excess Relative Risks (ERR), assumed to be normally distributed, as estimators of the DREF. The current authors were directly involved in either one or both of these studies and are well placed to examine how choices of methodology may influence evaluations of dose-rate effects on radiation-related cancer risks. A set of results from the paper by Shore et al. (2017) has been adopted here as a test set of data for applying different methods to explore this theoretical issue. In addition, many other meta-analysis methods were employed in work done for the Shore et al. (2017) paper, though not originally reported are now reported here, along with newly applied methods, to investigate further if there were any potential issues with the original choice of methodology used in Jacob et al. (2009), Shore et al. (2017).

## Materials and methods

### ***Methods originally applied in the two studies by ***Jacob et al. (2009)***, ***Shore et al. (2017)

The methods that were applied in the two studies of Jacob et al. (2009) and Shore et al. (2017) have been given already in detail in these papers. However, for completeness, they are summarized here. A comprehensive list of human radiation epidemiology studies with dose–response analyses of low dose-rate solid cancer data was compiled by literature review. For each low dose-rate study that was included in the meta-analysis, the published solid cancer excess relative risk (ERR) per Gy and the confidence interval (CI) were obtained directly from the publications. For each low dose-rate study included, a carefully matched ERR was calculated from the solid cancer ERR models fitted to the A-bomb Life Span Study (LSS) cohort. The matching was done on age-attained, age at exposure, sex proportion, incidence or mortality, solid cancer outcome type, and dose type, using the full LSS datasets and re-optimizing the LSS relevant models to be centered at the matched covariable values. Of particular note here is that no LSS sub-sets of data were used for this purpose, but the full cohort data (even the sex proportion was matched by applying weighted sex modifiers in the re-optimized LSS model). Then, the ratio of the ERR in low dose-rate cohorts to the ERR in the A-bomb Life Span Study (LSS) cohort was computed and the standard error of the ratio obtained by the standard method of Gaussian propagation of errors (Bevington and Robinson 2003) in Shore et al. (2017) and by simulation in Jacob et al. (2009) (but the simulation results were also confirmed with propagation of errors). The Gaussian propagation of errors method is also known as the Delta-method because of the application of derivatives and this latter terminology is applied hereafter.

The pooled, inverse-variance weighted mean ratio, *Q*, was calculated from the *q*_*i*_ study to LSS ERR estimates from all individual *i* studies (Table 1) under the basic premise that the average estimate calculated for the pooled study results is a better estimate than those provided by any of the individual studies. The variance of each q_i_ ratio was calculated from standard errors using the Delta-method and meta-analyses were done both with and without the assumption of heterogeneity of risk ratios (Sutton and Higgins 2008). Cochran’s *Q* statistic (and corresponding p value) method was applied to test for heterogeneity among study risk estimates and the DerSimonian–Laird method (DerSimonian and Laird 1986) was applied to account for heterogeneity between studies and for obtaining the overall variance on *Q*.

**Table 1:** Results for the excess relative risk (ERR) ratio, q_i_, the ratio of the individual study ERR/unit dose to matched LSS ERR/unit dose, and the standard error of q_i_, obtained with two different methods, Delta method (Bevington and Robinson 2003) as originally applied in Shore et al. (2017), or Fieller’s method (Fieller 1940)

Low dose-rate study			LSS		Combined estimates		
Cohort and reference	ERR/Gy	Std. Err	ERR/Gy	Std. Err	ERR ratio (*q*_*i*_) (study/LSS)	Std. Err Delta method	Std. Err Fieller's method
France, UK, US nuclear workers (Richardson et al. 2015)	0.47	0.185	0.3538	0.058	1.328	0.567	0.569
Japan nuclear workers (Akiba et al. 2012)	0.20	0.895	0.4551	0.144	0.439	1.972	1.998
Chernobyl liquidators (Kashcheev et al. 2015)	0.58	0.318	0.2282	0.056	2.542	1.529	1.543
Techa River (Schonfeld et al. 2013)	0.61	0.314	0.5288	0.056	1.154	0.606	0.607
Mayak workers (Sokolnikov 2015)	0.12	0.046	0.4271	0.066	0.281	0.116	0.116
Yangjiang high natural background (Tao et al. 2012)	0.19	1.253	0.4904	0.071	0.387	2.555	2.562
Rocketdyne (Boice et al. 2011)	− 0.20	0.893	0.2298	0.037	− 0.870	3.888	3.902
German U millers (Kreuzer et al. 2015)	0.27	1.419	0.3554	0.061	0.747	3.994	4.009
US nuclear power plant workers (Cardis et al. 2007; Howe et al. 2004)	0.51	1.696	0.4459	0.098	1.135	3.813	3.837
Canada nuclear workers (Zablotska et al. 2014)	− 1.20	1.832	0.3555	0.081	− 3.376	5.210	5.247
Port Hope (Zablotska et al. 2013)	0.12	0.439	0.2696	0.043	0.445	1.629	1.635
Sweden nuclear facilities (Cardis et al. 2007)	− 0.58	4.298	0.339	0.074	− 1.711	12.685	12.763
German nuclear power plant workers (Merzenich et al. 2014)	− 1.02	1.551	0.3182	0.076	− 3.207	4.934	4.971
Rocky Flats Plutonium facilities (Cardis et al. 1995)	− 1.63	1.295	0.2847	0.067	− 5.725	4.745	4.782
Belgian nuclear workers (Cardis et al. 2007; Engels et al. 2005)	− 0.59	4.152	0.3515	0.079	− 1.679	11.819	11.897
Finnish nuclear workers (Cardis et al. 2007; Auvinen et al. 2002)	174.00	544.729	0.3179	0.075	547.342	1718.319	1730.681
Spain nuclear facilities (Cardis et al. 2007)	1.02	7.830	0.3393	0.077	3.006	23.088	23.244
Australia nuclear workers (Cardis et al. 2007; Habib et al. 2005)	13.40	37.994	0.3835	0.076	34.941	99.315	99.833
Slovak nuclear workers (Gulis et al. 2003)	9.50	24.516	0.4008	0.101	23.703	61.457	61.968
Kerala high natural background (Nair et al. 2009)	− 0.13	0.265	0.336	0.059	− 0.387	0.793	0.796
Taiwan Co-60 contaminated flats (Hwang et al. 2008)	0.30	0.395	1.243	0.172	0.241	0.320	0.321
Korea nuclear workers (Jeong et al. 2010)	2.06	2.783	0.5575	0.113	3.695	5.048	5.075

for the calculations, the full precision available from the literature or from re-fitting the LSS models was applied

### Further methods applied here

Two further methods as suggested, for example, by Beyene et al. (2005), for error estimation of a ratio parameter are applied here. The first method is the Fieller's method (Fieller 1940) as a generic approach for a ratio parameter and this method was also suggested for this type of application by Little et al. (2021). The second method is the generalized linear modeling framework (McCullagh and Nelder 1989), considered here in the form of York regression (York 2004), which provides a basis for a useful re-parameterization of the current meta-analysis problem.

The Fieller’s method provides a novel way of expressing ratios as linear combinations of random variables and makes the computation of CIs or standard errors of ratios relatively simple, circumventing the theoretical issue of the ratio of two normal random variables having a theoretically unbounded variance. The full details are given in Fieller (1940), but for a modern description, suitable for swiftly understanding how to practically apply this method, and double checking the results via an inequality, see Beyene (2005). Details of the Fieller’s method are also given in Little et al. (2021). The Fieller’s method was applied here to obtain the standard errors and variances of each individual q_i_ ratio that goes into the meta-analysis of individual risk ratios.

**Fig. 1:**
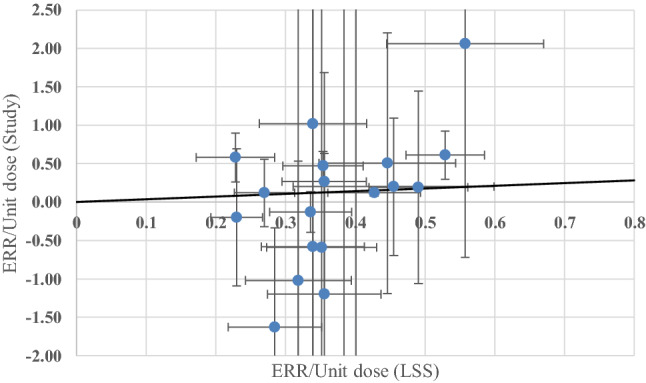
Result of the meta-analysis reformulated as a York regression with the best fit (all studies, solid black line), which is also the same if calculated with orthogonal distance regression. Note that some of the off-scale points from Table 1 were omitted to obtain an illustrative scaling here, but these outlying points were included in the fit. The points are given with standard error bars

Generalized linear modeling is widely applicable in several different scenarios with different distributional characteristics. The idea here, as broadly stated in Beyene et al. (2005), is that to estimate *i* quotients, a regression is carried out on the numerators and denominators of the quotients. Translated into the current problem of reformulating the meta-analysis here, regression is applied to the ERR/Gy values from the *i* low dose-rate studies as Y-axis variables with the ERR/Gy values from the LSS as X-axis variables (Fig. 1). For the situation in the current meta-analysis considered here, where each low dose-rate study ERR/Gy and each LSS ERR/Gy has a different uncertainty level, recommendations in the literature can be found for the unified regression algorithm of York (York et al. 1966, 1968, 2004). Two open-source programmed version of York regression was applied here, one implemented in the open-source R-statistical programming language, making use of the open-source R-function called York in the IsoplotR package and one implemented in the Numerical Recipes subroutine called “fitexy” (Press et al. 1992). In addition, orthogonal distance regression (Boggs and Rogers 1990) was applied as an alternative method for checking the results with the SciPy implementation (Virtanen et al. 2020).

Meta-analytic methods for assessing risk heterogeneity are currently developing, so heterogeneity was also evaluated using the several other methods cited in the following publications (DerSimonian and Kacker 2007) and in Table 2. Here again, use was made of the open-source R-statistical programming language functions for all but one of the meta-analysis methods given in Table 2 in the metafor package (Viechtbauer 2010) and the metrology package was used for the remaining meta-analysis method (Paule and Mandel 1982).

**Table 2:** Results for *Q* [the ratio of the study to LSS risks, i.e., the inverse of the dose-rate effectiveness factor (DREF)] and the standard error of *Q*, obtained with different methods

Method 1	Method 2	All studies Estimated *Q* (= 1/DREF)	All studies Std. Err. of *Q*	Excluding Mayak Estimated *Q*	Excluding Mayak Std. Err. of *Q*
Fieller's method (Fieller 1940)	Meta-analysis with fixed effects (Sutton and Higgins 2008)	0.3331	0.1040	0.5390	0.2314
Fieller's method	Meta-analysis with random effects (DerSimonian and Laird 1986)	0.3331	0.1040	0.5390	0.2314
^a^Delta-method (Bevington and Robinson 2003)	Meta-analysis with fixed effects (Sutton and Higgins 2008)	0.3331	0.1036	0.5393	0.2306
Delta method	Meta-analysis with random effects (DerSimonian and Laird 1986)	0.3331	0.1036	0.5393	0.2306
Delta method	Meta-analysis, restricted maximum-likelihood estimator (Viechtbauer 2005)	0.3368	0.1073	0.6003	0.2949
Delta method	Meta-analysis, DerSimonian–Laird estimator with adjustments (Knapp and Hartung 2003)	0.3331	0.077	0.5393	0.1695
Delta method	Meta-analysis, Hunter-Schmidt estimator (Hunter and Schmidt 2004)	0.3331	0.1036	0.5393	0.2306
Delta method	Meta-analysis, Hedges estimator (Hedges and Olkin 1985; Raudenbush 2009)	0.3331	0.1036	0.5393	0.2306
Delta method	Meta-analysis, Sidik-Jonkman estimator (Sidik and Jonkman 2005a, b)	0.0166	2.7112	− 0.0021	2.8980
Delta method	Meta-analysis, Empirical Bayes estimator (Morris 1983; Berkey et al. 1995)	0.3331	0.1036	0.5393	0.2306
Delta method	Meta-analysis (Paule and Mandel 1982)	0.3331	0.1036	0.5393	0.2306
Not required	York regression (York 2004) (in R-statistical software)	0.3488	0.1096	0.5925	0.2326
Not required	York regression, (York 2004) (in Numerical Recipes)	0.3488	0.0901	0.5928	0.2342
not required	Orthogonal distance regression (Boggs and Rogers 1990) (in SciPy)	0.3488	0.0860	0.5925	0.1765

The first column gives the method used to calculate the standard error of the ratio of the excess relative risk (ERR) ratio (*q*_*i*_) (study/LSS) and the second column gives either the type of meta-analytic method applied or the type of regression

^a^Note (for the third row of results): this is the combination of methods and the result of the meta-estimator of *Q* as originally reported by Shore et al. (2017)

### Test set of data for applying the further different methods

The full set of risks from the paper by Shore et al. (2017) has been adopted here, as it was in Little et al. (2021), as a test set for applying the different methods to. This is the set of ERR estimates from the 22 low dose-rate studies and their corresponding 22 LSS ERR estimates from Table 2 of Shore et al. (2017) and all associated uncertainties.

## Results

Table 1 gives the set of ERR estimates from the 22 low dose-rate studies and their corresponding 22 matched LSS ERR estimates from the Table 2 of Shore et al. (2017). The first column gives the low dose-rate study reference. Subsequent columns give: the ERR/unit dose for the low dose-rate study and the standard errors calculated from the published CIs; the ERR/unit dose from the matching LSS risk model central estimate and the standard errors obtained from the LSS model re-optimisations; the ratio of the two ERRs; the standard errors of the ratio calculated with the Delta method and the standard errors of the ratio calculated with Fieller’s method. It can be seen from the two last columns of Table 1 that the choice of method is not critical here, because very similar results are obtained with both methods, generally only differing in the second or third decimal places of the estimated standard errors, a difference of < 0.6% on average and only two studies had as much as a 1% difference. It follows from this result that, since the meta-analysis methods are inverse-variance weighted, the individual study weightings will also be very similar from the two methods.

Table 2 gives the Meta-estimator of Q (the aggregated ratio of the study to LSS ERR risks) as originally reported by Shore et al. (2017) and as newly calculated here by various meta-analysis methods and also applying the meta-analysis reformulated as a York regression or an orthogonal distance regression with zero intercept. The Mayak study had a mean external dose (354 mGy colon dose) much higher than any of the other studies, so Table 2 shows the results with and without that study. The first four entries in Table 2 confirm that the choice of method between the Delta method and Fieller’s method for calculating the standard errors of the ratios and therefore the weightings is not critical at all, because the Q values are the same to three or four decimal places and the standard errors are very similar (for all studies included, 0.1040 with Fieller’s method and 0.1036 with the Delta-method—a difference that would not usually be reported as noticeable in the literature which would normally only quote two decimal places here) and the standard errors are virtually identical. Specifically, the standard errors for Fieller’s method and the Delta method differed by 0.4%, 0.3%, 0.5%, and 0.4% for all mortality and incidence (M + I) studies, all M + I except Mayak, all M studies, and all M except Mayak, respectively. All other meta-analytic methods give consistent results except the Sidik–Jonkman estimator (Sidik and Jonkman 2005a, b) and the authors are currently investigating why this method gives different results. Table 2 also shows that York regression (York 2004) similarly produces results that are very close numerically to the various meta-analytic results. The best fitting line considering all studies, from York regression, which also corresponds to the line obtained with orthogonal distance regression, and most of the data points are shown in Fig. 1.

## Discussion

It has been thoroughly investigated here how different choice of methodology for evaluating dose-rate effects on radiation-related cancer risks may influence the overall results reported in a recent study (Shore et al. 2017). A set of results from the paper by Shore et al. (2017) has been adopted here as a test set of data for applying the many different methods described here that nearly all produced highly consistent results.

During the preparations for the first paper on DREF meta-analysis (Jacob et al. 2009), these potential theoretical issues associated with an unbounded variance of ratios were noted and thoroughly discussed (two statisticians were in this team). This is the reason why both papers (Jacob et al. 2009; Shore et al. 2017) are based on the meta-estimator of *Q* = 1/DREF, rather than estimating DREF directly. By estimating 1/DREF, one avoids the large and sometimes negative CIs, reported in some low dose-rate radiation epidemiology studies, occurring in the denominator of the risk ratio. By designing the methodology to have the matched LSS model results in the denominator, at least for the all solid cancer models, the CIs are well away from encompassing zero values. Furthermore, rather than basing the LSS risks on matched sub-sets of the LSS data, which could potentially have very wide CIs encompassing zero, the full LSS models were applied, using re-optimisation of the LSS risk models, so that they were centered at matching attained ages, ages at exposure, sex proportions, etc. The strategy of using the full LSS dataset modeled for the matching age and sex characteristics provides much more stable estimates of risks corresponding to the characteristics of each given LDR study than would estimates based on the selections of approximately matched sub-sets of the LSS data. That is, the full-data modeling approach appreciably reduces the uncertainty in the estimated LSS risk estimates, though there might be a minor trade-off in terms of estimation accuracy (i.e., unbiasedness).

The current analysis is intended to address methodological issues and does not imply that the authors recommend a particular value for the DDREF [see Shore et al. (2017) for discussions on DDREF relevant to radiation protection]. Table 2 shows that excluding the Mayak data affects the aggregated risk, but the conclusions relevant to the methodology do not change. It has been demonstrated here that consistent DREF estimators may be obtained using several complementary approaches. Furthermore, it has been shown that if the denominator of the ratio is estimated with sufficiently high precision, then the methods investigated and applied in this paper will produce very similar results.

The criticism involving the recalling of the well-known theoretical point—the ratio of two normal random variables has a theoretically unbounded variance—potentially causing some issues (Little et al. 2021) has been shown to be unfounded when aimed at the DREF published work cited and examined in detail here. However, for the purpose of reporting 95% CI of ratios for individual studies as done in Table 1 of Little et al. (2021), the use of Fieller-based CIs does make a small difference and more importantly a methodological improvement by removing the potential for critique due to theoretical issues.

The differences in corresponding central estimates in the results of Little et al. (2021) provided in Table 2 are not due to just the application of either the Delta method or the Fieller’s method (as their Table 2 caption and heading implies) and this will now be explained in detail. One reads in Little et al. (2021). “The difference made by calculation of central estimates and CI using either Eqs. (3) and (6) or Eqs. (8) and (11) are illustrated with a few examples, using data taken from Table 2 of the paper of Shore et al. (Shore et al. 2017).” Applying “Eqs. (8) and (11)” is just adopting the same methods as used in Shore et al. (2017), i.e., a meta-analysis based on one aggregate of 22 individual-study ratios where the 22 inverse-variance weightings were obtained using the Delta method. However, applying “Eqs. (3) and (6)” is just performing two meta-analyses, one meta-analysis to aggregate the risks from the 22 low- dose studies and one meta-analysis to aggregate the risks from the 22 matching LSS risks, where, in both meta-analyses, the 22 inverse-variance weightings are obtained directly from the published confidence interval results. In this latter regime, the ratio is formed from the aggregated study risk and the aggregated LSS risk [“Eq. (3)”], and the CI on this one ratio is calculated using the Fieller’s method. In the present paper, it was demonstrated practically (Tables 1 and 2) that it is also possible to apply equation “Eq. (8)” with “Eq. (6)” adapted for individual risk ratios, i.e., a meta-analysis based on aggregating 22 individual-study ratios where the 22 inverse-variance weightings are obtained using Fieller’s method—although this possibility appears not to have been considered in Little et al. (2021). The differences in corresponding central estimates of aggregated risks in the results of Little et al. (2021) provided in their Table 2 are due to the different aggregation methods. During the course of the work on the Shore et al. (2017) paper, the authors of that paper discussed aggregating the study risks and aggregating the LSS risk and taking the ratio, but decided against this method, because then the individual one-to-one matching information is lost. In the Shore et al. (2017) and Jacob et al. (2009) studies, a great deal of effort was made (using specially calculated dose conversion factors and re-optimized LSS models) to match each study risk with a specially computed LSS risk, because the individual study risks were for differing:

1. cancer outcome groupings for incidence or mortality: e.g., all solid cancers; all cancers except leukemia, all cancers excluding leukemia and alcohol-related cancers (oropharynx, esophagus, and liver); solid cancers except liver, lung, bone; and all solid cancers except liver;
2. reported doses: e.g., colon dose, skin dose, whole body or H_p_(10) dose, effective dose, stomach dose;
3. gender proportions;
4. ages at exposure;
5. attained ages;

therefore, it was considered very important, then as now, to retain the full matching information in the analysis. The matching, whether by (a) dividing individual study and LSS-matched risks or (b) keeping the risk matching via points in regression analysis, effectively adjusts for all other factors except the dose-rate effect, as far as possible given the study limitations. The present authors therefore: do not consider the use of aggregated study risk and the aggregated LSS risk [i.e., Eq. (3) of Little et al. (2021)] to be a methodological improvement, as the title and main text of Little et al. (2021) states; and recommend against this practice in this specific type of application. The sentence in the results section of Little et al. (2021) “The estimate of ERR_LDR_/ERR_LSS_ implied by the delta method for all studies excluding the Mayak data is 0.54 (95% CI 0.09, 0.99), whereas that implied by the Fieller method is 0.91 (0.28, 1.56) (Table 2),….” is open to potential misinterpretation, because this strong shift in central value from 0.54 to 0.91 is caused by leaving the exact one-to-one matching information out of the analysis, not by the change from using the Delta method to using the Fieller’s method. For illustration, on leaving out the one-to-one matching information and applying both methods to all studies excluding the Mayak data, one calculates a LDR/LSS ratio of ERRs [“Eq. (3)”] of 0.918 (95% CI 0.277; 1.572) for Fieller’s method and 0.918 (0.273; 1.563) for the Delta method; which indicates no difference in the central estimate of the ratio and only a slight difference in CI, in contrast to those comparisons given by Little et al. 2021. The contrast with the Little et al.’s results is because they did not use individual one-to-one matching. They reported differences between the Delta-method and the Fieller’s method of about 70% in the estimation of the LDR/LSS and about a 45% larger confidence bound for Fieller’s method (in their Table 2); whereas, with the individual-study matching used in the present work, there were no differences in the first three decimal places of the ERR ratios and differences in the widths of the ERR-ratio 95% confidence bounds of only 0.4%.

## Conclusion

It has been demonstrated here, in a practical way, that consistent DREF estimators may be obtained using several complementary approaches. Different choice of methodology for evaluating dose-rate effects on radiation-related cancer risks have been shown not to influence the overall results reported in a recent study (Shore et al. 2017) to any problematic degree. In fact, extremely similar results have been obtained with a wide variety of methods. Based on the results presented here, there is no convincing reason for believing that the previously reported results in Jacob et al. (2009) and Shore et al. (2017) should not stand as well-designed and thorough contributions to research in radiation protection. Furthermore and also based on the results presented here, it is recommended that for radiation protection purposes: meta-estimators of DREF should be calculated keeping the full one-to-one matching information in the analysis (e.g., as in Shore et al. 2017 paper); the Fieller’s method for calculating CIs on individual risk ratios is useful for reporting individual ratios; and a regression approach should be considered to be a useful and simple one-step approach.
